# Genes responding to water deficit in apple (*Malus* × *domestica* Borkh.) roots

**DOI:** 10.1186/1471-2229-14-182

**Published:** 2014-07-08

**Authors:** Carole Leavel Bassett, Angela M Baldo, Jacob T Moore, Ryan M Jenkins, Doug S Soffe, Michael E Wisniewski, John L Norelli, Robert E Farrell

**Affiliations:** 1 USDA, ARS, Appalachian Fruit Research Station, 2217 Wiltshire Road, Kearneysville, WV 25430, USA; 2 USDA, ARS, Dale Bumpers National Rice Research Center, 2890 HWY 130 E, Stuttgart, AR 72160, USA; 3 Department of Biology, Pennsylvania State University, 1031 Edgecomb Ave, York, PA 17403, USA; 4 Hagerstown Community College, 11400 Robinwood Drive, Hagerstown, MD 21742, USA

**Keywords:** Simulated drought, Fruit trees, Quantitative expression, Transcripts

## Abstract

**Background:**

Individual plants adapt to their immediate environment using a combination of biochemical, morphological and life cycle strategies. Because woody plants are long-lived perennials, they cannot rely on annual life cycle strategies alone to survive abiotic stresses. In this study we used suppression subtractive hybridization to identify genes both up- and down-regulated in roots during water deficit treatment and recovery. In addition we followed the expression of select genes in the roots, leaves, bark and xylem of ‘Royal Gala’ apple subjected to a simulated drought and subsequent recovery.

**Results:**

In agreement with studies from both herbaceous and woody plants, a number of common drought-responsive genes were identified, as well as a few not previously reported. Three genes were selected for more in depth analysis: a high affinity nitrate transporter (*MdNRT2.4*), a mitochondrial outer membrane translocase (*MdTOM7.1*), and a gene encoding an NPR1 homolog (*MpNPR1-2*). Quantitative expression of these genes in apple roots, bark and leaves was consistent with their roles in nutrition and defense.

**Conclusions:**

Additional genes from apple roots responding to drought were identified using suppression subtraction hybridization compared to a previous EST analysis from the same organ. Genes up- and down-regulated during drought recovery in roots were also identified. Elevated levels of a high affinity nitrate transporter were found in roots suggesting that nitrogen uptake shifted from low affinity transport due to the predicted reduction in nitrate concentration in drought-treated roots. Suppression of a *NPR1* gene in leaves of drought-treated apple trees may explain in part the increased disease susceptibility of trees subjected to dehydrative conditions.

## Background

Water is considered to be the most limiting environmental factor with regard to plant growth and maintenance. Furthermore, dehydration is a common component of other abiotic stresses, such as freezing, high temperatures, and salt stress. Loss of water not only affects plants in the short term, but can also weaken them, making them more susceptible to biotic and other abiotic stresses in the long term [1,2]. Plants have developed a variety of adaptations for ameliorating dehydrative stress. In one strategy, known as drought escape, the plant completes its lifecycle before arrival of the drier summer months. Another strategy, drought tolerance, results in the production of osmoprotectants, i.e. compounds that aid in preventing water loss from the cells or act to relieve the negative impact of the dehydrative stress on cellular components. A third mechanism, drought avoidance, allows plants to circumvent drought periods by morphological changes that allow plants to maintain high water status, for example, by encouraging deeper root penetration in the soil.

Another parameter related to plant water status is WUE which is a function of carbon utilization through photosynthesis and water loss through transpiration. In herbaceous plants there is often a tradeoff between suppression of photosynthesis and prevention of water loss, since WUE is closely tied to stomata function. For some annuals and perennials, increased WUE results in a measure of drought tolerance at the expense of growth and development [3,4]. On the other hand, some annuals maximize photosynthesis through increased stomatal conductance (defined as the rate of gas exchange through the stomata), thus lowering WUE. Although on the surface this seems counterintuitive, this strategy works because the plants ‘outgrow’ (complete their development) before the onset of seasonal droughts [5].

Regardless of which strategy(ies) a plant uses to survive, adaptation to drought requires complex interactions between anatomy, physiology and biochemistry, all of which are directly or indirectly under genetic control [6-8]. Studies examining genes in herbaceous plants that respond to dehydration have identified a number of common genes potentially related to drought resistance [reviewed in [9]. For example, in *Arabidopsis* several LEA genes (*Xero2*, *rd22*, *rab18*), metallothionein genes and a ripening-related protein gene have all been shown to respond to drought, as well as to other abiotic stresses [10,11]. These same genes in other plants (e.g., barley, chick pea, and rice) also show a strong response in transcript level when exposed to drought [12-14].

In woody plants similar studies have identified genes that appear to be significant for drought tolerance [15]. Transcriptional profiling was used to assess gene expression in poplar (*Populus euphratica*) subjected to a gradual drought treatment [16]. Since these studies were long term in contrast to studies with herbaceous plants, the percentage of genes expressed in response to drought was substantially lower. The expression of these genes may represent gradual adaptation, leading to acclimation to long term, moderate water deficit. In maritime pine (*Pinus pinaster* Ait.), Dubos and Plomion [17] identified 33 cDNA-AFLP fragments responding to polyethylene drought simulation, most of which were genes of unknown function. Genes which were down-regulated in roots included histone H2B, caffeic acid ortho-methyltransferase and a LEA protein, all of which have been shown to be up-regulated in other systems [11,14,18].

Suppression Subtractive Hybridization (SSH) has been used successfully to identify differentially regulated genes in a number of plant and animal systems [19,20]. Although different methodologies for assessing global gene expression have various strengths and weaknesses, SSH is known for its ability to identify low-abundance transcripts. In the current study we applied this method to identify genes up- and down-regulated in response to a simulated drought and at the end of a one week recovery period. Some of the genes have been previously described in other plants, but several genes crucial to metabolism or defense were unique to this study.

## Results and discussion

### Genes responding to simulated drought

**Figure 1:**
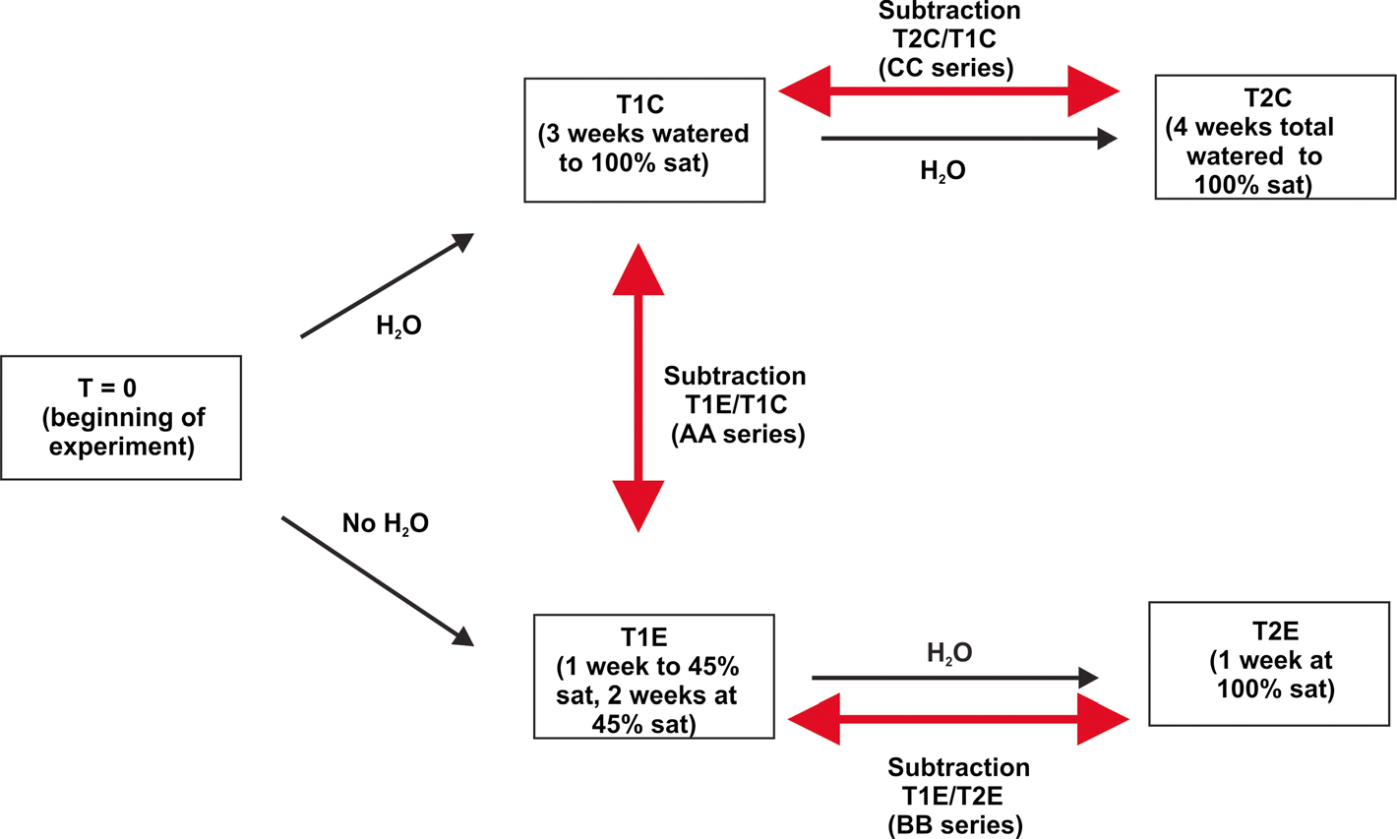
**Diagram of water deficit experiment conducted with ‘Royal Gala’.** A total of twenty-five trees were selected for the experiment. Five trees were sampled (leaves, bark and roots) at the beginning of the experiment (T = 0), after one week acclimation in the growth chamber. The controls, water deficit and recovery treatments are indicated in the boxes. Black arrows indicate how the experiment was conducted in time. Subtractions are indicated next to the red arrows showing the direction of subtraction, forward or reverse. For example, the forward subtraction between T1E and T1C (AAF) involves T1E cDNA as the tester and ten times concentrated T1C cDNA as the driver. Genes isolated from this subtraction represent those whose levels are upregulated in response to two weeks of water deficit treatment. In the reverse subtraction (AAR), T1C is the tester and T1E (ten times concentrated) is the driver. The reverse subtraction identifies genes downregulated in response to water deficit. The two controls (T1C and T2C) were subtracted to account for differences in gene abundance as a consequence of age (three weeks vs four weeks). This subtraction resulted in only a few sequences.

Genes whose mRNAs respond to drought have been identified and verified in a number of plant systems. We used SSH to identify genes in apple roots that were either up- or down-regulated by a simulated severe drought lasting two weeks (Figure 1). In addition, we identified genes whose expression changed after a week of water deficit recovery. Using bioinformatic tools we were able to design gene-specific primers for select genes from each treatment library to determine whether members of multigene families were identical or different in treatments where they appeared in more than one library (not shown). In this paper we focus on the results of our analysis of roots during water deficit and recovery (genes listed in Additional file 1).

Tables 1 and 2 contain a list of genes that were up- (AAF library) or down-regulated (AAR library) in response to water deficit treatment compared to well-watered controls run in parallel. Twice as many genes were identified in the library representing genes whose expression was elevated in response to drought compared to those that were down-regulated. Tables 3 and 4 list genes up-regulated after recovery from the drought treatment (BBR library) or elevated during drought treatment (BBF library) relative to recovery. Nearly three times as many genes were identified in the BBF library as in the BBR library. Because samples were taken after two weeks of drought, very early drought-responsive genes, including many transcription factors and signaling components, would not be expected to be identified in our libraries. One exception is the drought-responsive leucine zipper homeobox gene whose transcripts increased in drought-treated roots (manuscript in preparation). Since this gene is a close relative of the Arabiodpsis AtHb7 and AtHb12 genes which are also drought-induced [21], its elevated presence two weeks after the beginning of the drought period may be indicative of a role in maintenance of the drought response.

**Table 1:** **Sequences up-regulated after two weeks of simulated drought (T1E tester vs T1C driver)**^**1**^

**SSH ID**	**Gene description**	**SSH ID**	**Gene description**
Contig1AAF	Major latex protein	C01AAF	Ω-hydroxypalmitate-O-feruloyl
Contig8AAF	Major latex protein		transferase-like
A09AAF	Major latex protein		
Contigs2/3AAF	Metallothioneine^2^	C05AAF	60S ribosomal protein L38
	Metallothioneine		
A11AAF	Metallothioneine fragment		
D09AAF	Metallothioneine		
Contig 4AAF	Putative PIP2-5 homolog^3^	C08AAF	Phospholipase C3-like
Contig5AAF	Reticulon B2-like	C09AAF	Mitochondrial import receptor
A06AAF	ATP citrate synthase	C10AAF	SAUR family protein
A10AAF	Adenosylhomocysteinase	D05AAF	NPR1
A12AAF	Actin	D11AAF	Cyclophilin
B02AAF	Mal d 1^4^	D12AAF	Leucine zipper homeobox
B03AAF	Bypass 1		

^1^There were 8 unidentified or hypothetical genes in the AAF subtraction.

^2^Contig2_AAF is most closely related to *Arabidopsis* MT2B and Contig3_AAF is most closely related to MT2A.

^3^Plasma Membrane Intrinsic Protein. Nearly identical to the PIP2 in the BBF subtraction.

^4^This clone is identical to the mal d 1l translated product [61].

**Table 2:** **Sequences down-regulated after two weeks of simulated drought (T1E tester vs T1C driver)**^**1**^

**SSH ID**	**Gene description**
Contig1AAR	5-methyltetrahydropteroyltriglutamate homocysteine methyltransferase
Contig7/B11AAR	Germin-like gene
Contig8AAR	Mal d 1^2^
Contig9/10AAR	Putative PIP2-4 homolog^3^
B04AAR	α-L-fucosidase 2
B05AAR	LEDI-3-like protein
D01AAR	WD-repeat protein 5
D07AAR	Mavicyanin^4^

^1^There were 14 unidentified and 3 hypothetical genes from this subtraction.

^2^This EST differs considerably from Mal d 1l [61] and is most closely related to Mal d 1.03A01 [62].

^3^Appears to be a different family member from the PIPs in the AAF and BBF subtractions.

^4^A blue copper protein.

**Table 3:** **Sequences downregulated after a week of recovery from simulated drought (T2E driver vs T1E tester)**^**1**^

**SSH ID**	**Gene description**	**SSH ID**	**Gene description**
Contig5BBF	Protease inhibitor-like	F05BBF	Programmed cell death protein
E03/E12/F08/G05/H08BBF	Metallothionein^2^	F10BBF	Histone H2B
E05BBF	High affinity nitrate transporter	G01/H09BBF	Mal d 1
E07BBF	Major latex protein	G09BBF	High molec weight HSP^4^
E08BBF	Isoflavone reductase	H01BBF	Copper chaperone
E10BBF	Putative PIP2-5 homolog^3^		
E11BBF	DNA binding protein-like	E06BBF	SAT5

^1^There were six unidentified and one hypothetical genes from this subtraction.

^2^F08BBF is related to *Arabidopsis* MT2B; G05BBF is related to MT2A; the remaining three ESTs could represent as many as three different metallothioneine family members.

^3^See note 3 Table 1.

^4^Probable HSP70 family member.

**Table 4:** **Sequences upregulated after a week of recovery from simulated drought (T2E tester vs T1E driver)**^**1**^

**SSH ID**	**Gene description**
Contig1/G12BBR	Cysteine protease
G11BBR	Ankyrin repeat protein-like
H10BBR	Glutathione S transferase
Contig2/H11BBR	Early nodulin 16 precursor-like^2^
H04BBR	Blue copper protein-like

^1^There were five unidentified and one hypothetical genes from this subtraction.

^2^Binds copper.

In three of the SSH libraries copper-binding proteins were identified. The H01BBF sequence (up-regulated in drought) matched (E value: 9e-33) a copper chaperone from *Fragaria vesca* (ATOX1-like; XP_00408552). Three putative copper binding proteins were also identified in the BBR (down-regulated) subtraction: H11BBR and Contig2BBR aligned with an early nodulin16 precursor (E values: 1e-15 and 4e-17, respectively) from *Ricinus communis* (XP_002527193), and H04BBR aligned with a predicted copper binding protein from *Prunus persica* (E value: 1e-18; EMJ04613). In addition, clone D07AAR (down-regulated) aligned with another copper binding protein, mavicyanin, from *Ricinus communis* (EEF36698). With the exception of H01BBF, all of these genes appear to be down-regulated in apple roots in response to drought. When cellular copper is limiting, copper chaperones are generally required [22,23]. If Cu^+2^ uptake is reduced by drought treatment, the up-regulation of H01BBF in apple roots would be consistent with previous studies and may reflect its function as a member of the ATOX copper chaperone family, namely the intracellular delivery of copper to the secretory pathway [23].

Three plasma membrane-intrinsic protein (PIP or aquaporin) ESTs were found in roots (Tables 1, 2 and 3). When the derived amino acids were analyzed with BLASTp against the *Arabidopsis* genome, the two ESTs from drought up-regulated libraries (Contig4AAF and E10BBF) were found to be most closely matched with *Arabidopsis* PIP2-7 and PIP2-5, respectively (E values: 2e-69 and 1e-103). The AAR library EST (Contig 9) was more closely related to *Arabidopsis* PIP2-4 (E value: 2e-27). Comparison of the ESTs with each other indicated that the AAR and AAF sequences both aligned with the BBF sequence, but with no overlap between them. In addition, most of the amino acid differences between AAR and BBF were non-conserved substitutions, as opposed to the conserved differences seen between the AAF and BBF sequences. Taken together the results suggest that these genes in fact represent different family members.

Since abiotic stress affects a number of critical plant processes, the identification of genes representing a variety of cellular functions in response to drought is to be expected. In combining sequences up-regulated in drought, we included genes from both the AAF and BBF libraries. In both cases the total number of up-regulated genes in response to drought exceeded the number of down-regulated genes by nearly two-fold. Many of the identified genes have been previously reported in other plant systems subjected to various types of drought stress [7,21]. For example, metallothionein and related genes are elevated under water deficit conditions in rice, chick pea, and *Arabidopsis*[11,13,14]; likewise, PIPs and mal d1 have also been commonly associated with dehydration responses.

### Analysis of specific genes

In order to confirm treatment differences correlated with SSH and affirm the integration of genes responding to water deficit, we conducted an in-depth analysis of three genes whose role under drought conditions has not been well characterized at the molecular level. These genes included ESTs encoding a high affinity nitrogen transporter (*MdHAT2.4*), an outermembrane mitochondrial import receptor subunit (*MdTom7.1*) and a gene (*MpNPR1-2*) associated with regulons involved in both systemic acquired and basal resistance to biotic stress.

#### High affinity nitrate transporters

E05BBF is an EST isolated from the root BBF library (up-regulated in drought relative to recovery) and was identified as a high affinity nitrate transporter. In *Arabidopsis* the high affinity nitrate transporters are represented by seven genes: *NRT2.1-2.7. AtNRT2.1* and *AtNRT2.2* are the primary genes responsible for transport of nitrate under low nitrogen availability and appear to be inducible, since a mutant lacking both genes fails to achieve nitrate transport levels similar to the wild-type [24]. However, a very high-affinity component is still active in this mutant, and it is thought that this activity corresponds to *AtNRT2.4*, the most inducible gene under limiting nitrate conditions.

The full length apple gene (MDP0000239537) represented by the E05BBF EST is on chromosome 11. There are three other full length apple genes closely related to E05BBF (Additional file 2). Of these, MDP0000266497 is most like E05BBF and appears to be related to *AtNRT2.5* (73% amino acid identity). The remaining two genes are most closely related to *AtNRT2.7*. MDP0000266497 is located on chromosome 13, whereas MDP0000131368 and MDP0000201530 are located on chromosomes 9 and 17, respectively. All of these predicted proteins are members of the major facilitator superfamily (transport of small solutes across membranes) and possess a nitrogen transporter domain as well [25].

MDP0000239537 (E05BBF; *MdNRT2.4*) has significant homology to both *AtNRT2.1* and *AtNRT2.4* (Table 5). In a recent study of *Malus hupenensis* (Pamp.) Rehder [26], a full length nitrate transporter gene (designated *MhNRT2.1*) was found to share 98.9% homology with MDP0000239537, whereas a second gene (designated *MhNRT2.5*) shared 98.6% amino acid identity with MDP0000266497.

**Table 5:** **Comparison of the high affinity nitrate transporter from the BBF subtraction with the class 2 high affinity nitrate transporter family from *Arabidopsis***^**a**^

***Arabidopsis *** **gene ID**	**Tot score**^**b**^	**Coverage**^**c**^	**E value**^**d**^	**Maximum identity**^**e**^
NRT2.1	893	100%	0	80%
NRT2.2	817	96%	0	76%
NRT2.3	792	99%	0	73%
NRT2.4	904	100%	0	81%
NRT2.5	611	91%	2e-179	61%
NRT2.6	790	99%	0	73%
NRT2.7	441	80%	4e-128	50%

^a^There was no significant similarity between the BBF sequence and the *Arabidopsis* class 1 or class 3 genes.

^b^The total score of an alignment is calculated as the sum of substitution and gap scores.

^c^The percentage of query residues that align with the subject residues.

^d^The Expect value represents the number of different alignments that is expected to occur in a database search by chance. Value of 0 means the expect value was less than e-179.

^e^The extent to which two amino acid sequences have the same residues at the same positions in an alignment.

**Figure 2:**
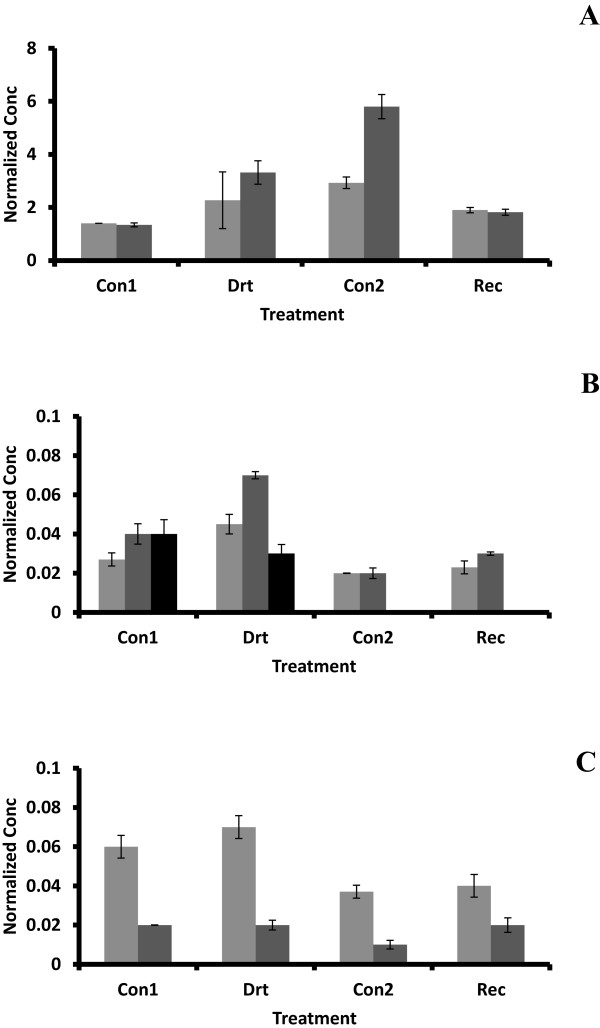
**Quantitative analysis of *MdNRT2.4 *expression during water deficit and recovery.** Expression was assessed by quantitative RT-PCR as described in Methods. Concentrations were determined by normalization with TEF2, and the results from two independent experiments are shown. Standard error bars are indicated on the columns. **A:** Root; **B:** Bark and Xylem; **C:** Leaf. Con1 and Con2: well watered control plants for water deficit treatment and recovery, respectively, Drt: water deficit treatment and Rec: recovery. Light gray columns = 2005 experiment; dark gray and black (xylem) columns = 2008 experiment.

To obtain accurate estimates of the differences in abundance between the drought treatment and recovery, RT-qPCR was performed on all the treatments from roots, as well as samples taken from leaves, bark and xylem subjected to the same conditions (Figure 2). Expression of *MdNRT2.4* in leaves was not significantly different in drought-treatment vs. controls (Figure 2C), although the additional week of recovery resulted in an overall decline of transcript abundance for reasons not completely clear. Orsel et al. [25] reported that *AtNRT2.4* was substantially inducible in low concentrations of KNO_3_, whereas Okamoto et al. [27] observed repression of *AtNRT2.4* levels in both roots and shoots exposed to 0.5 mM Ca(NO_3_)_2_ after nitrogen deprivation. In our study the levels of the *MdNRT2.4* transcript from plants under water deficit were 212% of the control in roots and 167% of control in bark; in both organs, recovery resulted in a return to control levels (96% and 115% of controls, respectively). Since nitrogen uptake is by mass flow of water from the soil to the root [28,29], lower nitrogen levels would be expected in roots of plants under water deficit. In wheat nitrogen-use efficiency was increased by water deficit and diminished in response to increasing concentrations of applied nitrogen [30]. Our observations regarding *MdNRT2.4* expression are consistent with these reports and may provide a novel avenue for exploring drought resistance, since links between nitrogen deficiency, water deficit response and ABA/stomatal function have been previously established [31].

Analysis of approximately 700 bases upstream of the translation start codon of MdNRT2.4 identified several *cis*-elements related to stress or hormone response (Additional file 3). Two TATA boxes representing RNA PolII binding sites were found, one approximately 100 bases from the translation start site. There were no consensus G-box elements in either promoter [32,33]. Elements similar to G-box abscisic acid response elements (ABREs) [34] were present in both the *Arabidopsis* and apple *NRT2.4* promoters. However, *NRT2.4* is not likely to respond to drought via ABA induction because the core sequence in both G-box elements does not end in cytosine (C_+4_) which is essential for ABA induction [35]. On the other hand, a consensus C-repeat binding element linked to both cold and drought response was within a functional distance of the first TATA box in the apple *NRT2.4* promoter. The second TATA box is further upstream and is linked to several MYC binding sites, as well as a wound-inducible element [36] and an element associated with hypoosmolarity responsiveness [37]. Both TATA elements could be functional under different regulatory regimes. Mapping transcripts originating from this promoter would determine whether or not both TATA elements are functional. Many of the elements identified in the *MdNRT2.4* promoter were absent in the *Arabidopsis* promoter, and there were considerably fewer MYB binding sites (CANNTG) in the *AtNRT2.4* promoter (data not shown).

#### Mitochondrial import translocase subunit (TOM)

The mitochondrial import complex (TOM for Translocase of the Outer Membrane and TIM for Translocase of the Inner Membrane) is extensively conserved in eukaryotes and typically contains 13 TIM and seven TOM subunits [38]. The TOM subunits consist of two receptors (TOM70 and 40) that interact with the pre-protein and five subunits that compose the translocation channel (TOMs 22, 20, 7, 6, and 5). Only three outer membrane proteins are ubiquitous among eukaryotes: TOM40, TOM22 and TOM7 [39].

An EST (C09AAF) was identified in the drought-treated root subtraction encoding a homolog to the TOM7 subunit. RT-PCR results indicated that C09AAF levels were lower in the drought-treated plants than in the controls. This is not consistent with this sequence having been obtained from the AAF subtraction representing up-regulated sequences. Close inspection of the PCR reaction products indicated marked similarity in band intensity between the treatments and also revealed an unpredicted, higher molecular weight band that was amplified, suggesting that a close relative might be interfering with primer hybridization (data not shown).

BLASTn alignment of C09AAF against the apple genome found three closely related genes on different chromosomes, one of which (MDP0000023053) corresponded perfectly with the coding sequence of C09AAF. The three apple *TOM7* genes are found on chromosomes 12, 13 and 16. MDP000023053 (designated as *MdTOM7.1*) is located on chromosome 13. Several other significant BLASTn hits were also noted, but these sequences maybe pseudo-genes, as the ATG codons are altered. Conservation between the apple derived protein sequences and two *Arabidopsis* TOM polypeptides is confined mainly to the TOM7 domain characteristic of the superfamily (Additional file 4).

**Figure 3:**
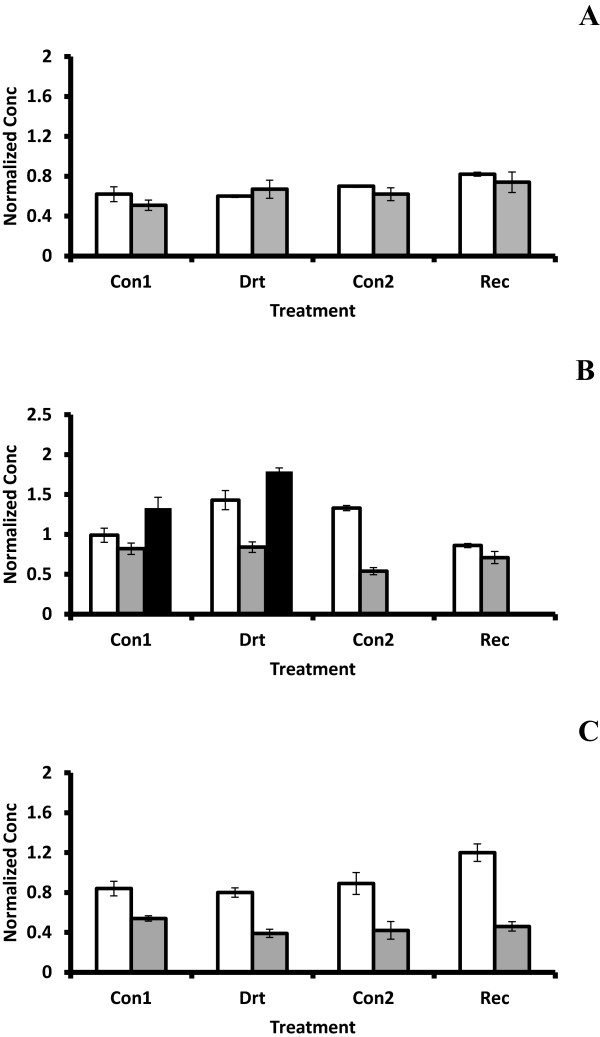
**Quantitative analysis of *MdTOM7-1 *expression during water deficit and recovery.** Expression was assessed by quantitative RT-PCR as described in Methods. Concentrations were determined by normalization with TEF2, and the results from two independent experiments were averaged. Standard error bars are indicated on the columns. **A:** Root; **B:** Bark and Xylem; **C:** Leaf. Con1 and Con2: well watered control plants for water deficit treatment and recovery, respectively, Drt: water deficit treatment and Rec: recovery. Light gray columns = 2005 experiment; dark gray and black (xylem) columns = 2008 experiment.

RT-qPCR primers were designed to eliminate any possible contribution from the other two related genes in the RNA populations. Testing of this primer set indicated that only one product was obtained (not shown). This primer set was then used for RT-qPCR analysis and the results are shown in Figure 3. In bark, *MdTOM7.1* was moderately elevated under drought conditions compared to its control. On the other hand, *MdTOM7.1* in roots and leaves was not appreciably different from the well watered controls and did not decline to control levels during the recovery phase as observed in bark. This is supported by analysis of the *MdTOM7* promoters where no ABRE or DRE sequences were found in the first 800 bases upstream of the ATG codon (Additional file 5). In contrast both elements were present just upstream of the TATA box in *MdTOM7.2* (MDP0000694615). In apple leaves expression of *MdTOM7.1* was similar to the expression of At5g41685 [*TOM7-1*] and At1g64220 [*TOM7-2*] (Gene Expression Omnibus, NCBI) in *Arabidopsis* whole plants treated with salt or exposed to 0 °C, showing little or no change compared to controls. However, both *Arabidopsis* genes show greater accumulation in the polysomal fraction of plants exposed to dehydration treatment, relative to control polysomes or the non-polysomal fraction of controls and treated plants [40], indicating that regulation of TOM7 may not be solely transcriptional. Based on these observations and the presence of stress-responsive elements in the promoter of *MdTOM7.2*, it would be of interest to examine expression of this gene in the same RNA populations from the organs of drought-treated apple to determine if it indeed responds to water deficit treatment.

TOM7 binds to TOM40 in the outer mitochondrial membrane where it is thought to modulate pore formation and might be expected to play a role during drought stress. The fact that drought did not appear to alter *MdTOM7.1* transcript levels in apple or *Arabidopsis* suggests that regulation at another level may be more important to plant *TOM7.1* or that drought treatment alters another component of the mitochondrial import complex (possibly *MdTOM7.2*) to allow continual mitochondrial functioning in plants under stress.

#### Nonexpressor of pathogenesis related genes (MpNPR1-2)

**Figure 4:**
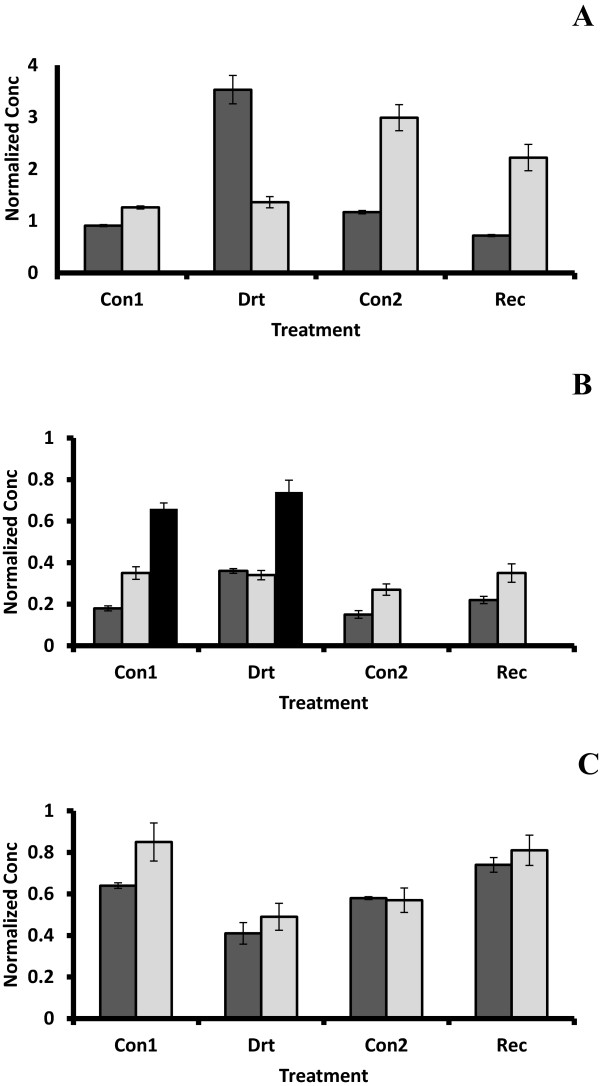
**Quantitative analysis of MpNPR1-2 expression during water deficit and recovery.** Expression was assessed by quantitative RT-PCR as described in Methods. Concentrations were determined by normalization with TEF2, and the results from two independent experiments were averaged. Standard error bars are indicated on the columns. **A:** Root; **B:** Bark and Xylem; **C:** Leaf. Con1 and Con2: well watered control plants for water deficit treatment and recovery, respectively, Drt: water deficit treatment and Rec: recovery. Light gray columns = 2005 experiment; dark gray and black (xylem) columns = 2008 experiment.

An EST (D05AAF) encoding *MpNPR1-2*[41] was isolated from the library containing genes up-regulated in response to drought (Table 1). Quantitative assessment of *MpNPR1-2* expression in roots, bark and leaves of drought-treated apples indicated that its mRNA was elevated nearly four times in drought-treated roots over control roots in the 2008 experiment, although in an earlier experiment there was essentially no difference (Figure 4A). A smaller increase was noted in drought treated bark and xylem (Figure 4B), but surprisingly drought treatment lowered its expression in leaves from both experiments (Figure 4C).

A previous study of NPR expression in apple identified three NPR1 genes [41]. but only *MpNPR1-1* was induced with BTH (Benzo(1,2,3)thiadiazole-7-carbothioic acid) treatment to induce systemic acquired resistance (SAR), suggesting that this may be the ortholog to the *Arabidopsis* gene, *AtNPR1. AtNPR1* encodes a protein with ankyrin repeats that binds to transcription factors of the TGA subfamily of basic leucine zipper proteins [42,43]. Traditionally, NPR1 has been associated with salicylic acid (SA)-linked systemic acquired resistance affecting a broad spectrum of pathogens, including fungi, bacteria and viruses. A recently described role for cytoplasmic NPR1 in jasmonic acid (JA) suppression has been reported [44], as well as an association with basal defense response [42,45]. Expression of *AtNPR1* is generally constitutive showing only a modest (two time) induction by SA [46].

Cross-talk between signaling pathways has been known for some time. For example, drought-stressed plants are generally more susceptible to pathogen attack than unstressed plants [47,48]. A study in rice revealed a connection that may be related to the JA suppression role of NPR1 [49]. In this study, constitutive expression of the *Arabidopsis AtNPR1* gene in rice leaves conferred resistance to several fungal pathogens and one bacterial pathogen by ‘priming’ the SAR pathway. Interestingly, the same transgenic lines resistant to the pathogens were more sensitive to drought and salt treatments. These results correlated with reduced expression (both in abundance and timing) of some key genes associated with abiotic stress, e.g. *Rab21*. Our results appear to corroborate suppression of *NPR1-2* in leaves under drought stress with studies indicating increased susceptibility of stressed plants to different pathogens. Most interesting is the observation that *MpNPR1-2* is significantly elevated in roots at the end of a two-week, continuous water deficit treatment. These results suggest a role for *MpNPR1-2* in root-specific protection against soil-borne pathogens during drought stress.

## Conclusion

We have identified apple root genes that respond to a two week, moderately severe simulated drought and to a one week period of recovery. Most of the genes identified have been previously reported to be drought- or stress-responsive in other plants. Three genes not previously associated with root response to drought were further characterized. Two genes, *MdNRT2.4* and *MpNPR1-2* were shown by quantitative RT-PCR analysis to be up-regulated in apple roots subjected to drought. The third gene, *MdTOM7.1* was not appreciably expressed in response to drought treatment.

The results from two independent experiments demonstrate that drought treatment increases expression of a high-affinity nitrate transporter (*MdNRT2.4*) which is consistent with previous research associating drought with nitrogen deficiency. Our results from the 2008 experiment also suggest that *MpNPR1-2* may have a defense role in roots, since its up-regulation in this organ in response to abiotic stress has not been previously reported. Finally, a reproducible decrease in *MpNPR1* in leaves in association with drought treatment may explain why many plants under abiotic stress are reported to be more susceptible to pathogen attack. Further analysis of these gene families may identify altered functions or expression for individual family members arising as a requirement for adaptation to the varying environmental conditions that most perennial plants face.

## Methods

### Plant material

Apple plants of a single genotype (*Malus × domestica* cv. Royal Gala) were initially propagated by asexual *in vitro* shoot proliferation (clonal replication) culture at the USDA-ARS-NAA-AFRS facility (Kearneysville, WV) as per Norelli et al. [50] and Ko et al. [51], with root induction as per Bolar et al. [52]. After rooting and establishment, the young trees were potted in standard ten-inch nursery pots containing Metromix 310 (composition: horticultural vermiculite, Canadian Sphagnum peat moss, processed bark ash, composted pine bark, and washed sand [Scotts – Sierra Horticultural Products Co., Marysville, OH]) and transferred to a glasshouse. The trees were grown under supplemental lighting to maintain day length at 16 h, and a temperature range of 20–35°C. Trees were watered daily, with weekly application of nutrient solution (MiracleGro) and with supplemental application of Osmocote (Scott’s Miracle-Gro Products; 19-6-12 N-P-K) every two months at the indicated rate of 10 g/pot. Trees were in the glasshouse for a total of 8 months, with final caliper measurements ranging from 0.5 cm to slightly more than 1.0 cm, and heights varying from 1 to 2 m.

Twenty-five trees were placed in a Conviron PGV36 growth chamber (Conviron) at 25°C day (16 h)/18°C night (8 h) with light at 500 μmol photons m^−2^ s^−1^ PPFD and acclimated for one week, after which twenty trees were selected for experimentation. Water deficit was imposed essentially as described by Artlip and Wisniewski [53]. The experiment is diagrammed in Figure 1. Water was withheld from ten trees until the pot masses were at 45% of the saturated mass and maintained at this level for two weeks without added fertilizer. Ten control trees were maintained at the saturated mass by daily watering without fertilizer. After two weeks of water limitation, roots, bark and leaves were harvested from five water deficit-treated trees and five well watered controls. The remaining five of the water deficit-treated trees were watered to saturation for one week, along with the five remaining controls. No fertilizer was applied during the experiment. The samples from each individual organ and treatment were pooled, immediately frozen in liquid N_2_ and stored at -80°C. Harvested leaves were taken from leaf positions 7 through 12 (counting down from the youngest visible leaf at the apex), and typically measured from 6.5 to 9.0 cm in length. These leaves are considered to be at or near full expansion. To avoid complication due to circadian rhythm effects on expression, samples were taken at the same time of day approximately 8 h after ‘lights on’ in the growth chamber.

### Suppression subtractive hybridization

Total RNA was isolated from each organ by a method reported by Artlip et al. [54]. The RNA was treated with DNase according to the manufacturer’s (InVitrogen) recommendation and tested for DNA contamination by PCR prior to use in downstream applications. Suppression subtractive hybridization was performed as described previously [20] using the Super SMART method (Clontech, Palo Alto, Calif.) for cDNA synthesis and following the manufacturer’s protocol for subtraction hybridization (Clontech).

### PCR analyses

Leaves, roots and bark subjected to the two week drought treatment and one week recovery were collected from two independent experiments conducted in 2005 and 2008. Total RNA was extracted, DNased and quality-assessed on agarose gels. cDNA was synthesized with the Advantage RT kit following the manufacturer’s directions (Clontech).

Primers for RT-qPCR (Additional file 6) were designed with Primer 3 Plus software [55] and tested against genomic DNA for quality assurance. Each primer pair was used to prime RT-qPCR reactions in order to quantify gene expression in different organs. The qPCR reactions were conducted using a kit containing all the reagents (Life Technologies, Applied Biosystems, Grand Island, NY), and the reaction parameters were as follows: 95°C 5 min, followed by 35 cycles of 95°C 1 min, 60-65°C 1 min, 72°C 1 min and a final extension of 72°C for 10 min. Primers for a translation elongation factor (TEF2) were used as an internal control for the qPCR experiments [56]. The relative standard curve method was used to analyze the data.

### Bioinformatic analyses

Sequence data and GenBank accession numbers are included in Additional file 1. Remaining vector sequences were manually identified and checked with VecScreen at the National Center for Biotechnology Information (NCBI). Each library was analyzed with the Cap3 Assembly program (Iowa State University) to obtain a unigene file for each forward and reverse subtraction. The unigene files were analyzed with BLASTx, BLASTn and/or tBLASTx (NCBI) to identify specific sequences. Alignments were performed with Cobalt (NCBI) or ClustalW using the BLOSUM matrix. Apple promoter sequences were identified at Genome Database for Rosaceae (http://www.rosaceae.org/) from the whole genome sequence of ‘Golden Delicious’. Promoters were defined as the first one thousand bases upstream of the translation start codon. *Cis*-elements were identified with PLACE [57], PlantPAN [58] and PlantCare [59,60].

## Availability of supporting data

GeneBank accession numbers are included in Additional file 1.

## Abbreviations

AAF and AAR: Sequences up- (AAF) or down- (AAR) regulated after two weeks of simulated drought; ABRE: ABA response element; BBF and BBR: Sequences up- (BBR) and down- (BBF) regulated after a week of recovery from simulated drought; CCF and CCR: Sequences up- (CCF) and down- (CCR) regulated between treatment weeks 2 and 3 in trees not exposed to drought; DRE: Drought response element; EST: Expressed sequence tag; LEA: Late embryogenesis abundant; MDP#: *M. × domestica* predicted coding region (see http://www.rosaceae.org); RT-qPCR: Quantitative reverse transcription-polymerase chain reaction; SSH: Suppression subtractive hybridization; WUE: Water use efficiency.

## Competing interests

There are no competing interests.

## Authors’ contributions

CLB: designed the experiments, conducted RT-qPCR analyses, and performed in-depth bioinformatic analyses of genes AMB: conducted unigene assembly, provided initial bioinformatic analyses of subtraction results. JTM and RMJ: performed quality assurance analyses of DNA and primers and conducted semi-quantitative PCR reactions. DSS: performed semi-quantitative and quantitative PCR reactions. MEW: contributed to the experimental design and interpretation of data. JLN: contributed to the interpretation of data and preparation of manuscript. RJF: conducted SSH experiments and performed quality control experiments on SSH results.

## Authors’ information

Jacob T. Moore and Ryan M. Jenkins are students at Pennsylvania State University; Doug S. Soffe was a student intern from Hagerstown Community College.

## Supplementary Material

Additional file 1 **Sequences up- (AAF) or down- (AAR) regulated after two weeks of simulated drought; Sequences up- (BBR) and down- (BBF) regulated after a week of recovery from simulated drought; Sequences up- (CCF) and down- (CCR) regulated between treatment weeks 2 and 3 in trees not exposed to drought (recovery control, subtractions T2C/T1C in Figure** 1**).** DNA sequence of unigenes and sequence annotation. Note: sequences not accepted by GenBank dbEST included sequences less than 200 nts, sequences encoding hypothetical or unknown proteins, mitochondrial and chloroplast DNA, and ribosomal DNA. Sequences making up contigs may not be assigned an accession number if one of more of the sequences is less than 200 nts. Click here for file

Additional file 2 **Alignment of full length apple High Affinity Nitrate Transporter genes.** MFS: major facilitator superfamily domain and NNP: nitrate/nitrite porter domain defined within alignments. Click here for file

Additional file 3 **Comparison of HAT2.4 promoter region from apple and *Arabidopsis.*** *Cis*-elements identified by PLACE [57] and PLANTCare [59-69] are shown for the first 700 bases upstream of the translation start (atg) [63-70]. Click here for file

Additional file 4 **Alignment of apple TOM7 predicted polypeptides.** Predicted polypeptides for the three apple TOM7 polypeptides and the EST isolated from drought-treated roots are aligned with the two predicted *Arabidopsis* TOM7 polypeptides [71]. Click here for file

Additional file 5 **MdTOM7 promoter regions.** Sequences approximately 800 bases upstream of the translation start (atg) were analyzed by PLACE, PlantPAN and PlantCare [72][67]. Click here for file

Additional file 6 Sequences of primers used for RT-qPCR are listed. Click here for file
