# Supplementary material for: Genes responding to water deficit in apple (Malus × domestica Borkh.) roots
Source: BMC Plant Biol. 2014 Jul 8;14:182. doi: 10.1186/1471-2229-14-182 (PMC4110548; doi:10.1186/1471-2229-14-182)
Supplement: Additional file 2 — Alignment of full length apple High Affinity Nitrate Transporter genes. MFS: major facilitator superfamily domain and NNP: nitrate/nitrite porter domain defined within alignments. [file 1471-2229-14-182-S2.docx]

MDP0000266497 MEVESRVMEPGSPKFALPVDSDHKATEFRLFSIAKPHMRAFHLSWFSFFLXFVSS

MDP0000239537 MADSEGEPGSSMHGVTGREQTFAFSVASPIVPTDTTAKFDLPVDSEHKAKVFKLFSLANPHMRTFHLSWISFFTCFIST

MDP0000201530 MFALPVDADQKATELRLFSIAPPHMLAFHLAWLSLFSNFFST

MDP0000131368 MFELPVDADQKATELRLCSIAPPHMLAFHLAWLSLFSNFFST

MDP0000266497 FAAPPLIPIIRDNLNLTASDIGNAGIASVSGAVFARIAMGTACDLFGPRLASATLILLTAPAVYFTAIASSPMSFLLVR

MDP0000239537 FAAAPLVPIIRDNINLTKQDIGNAGVASVSGSIFSRLVMGAVCDLIGPRYGCAFLIMLSAPTVFCMSFVADAGGYIAVR

MDP0000201530 FSIPPLLAVIRDDLNLTDTDTGHAGTAXFLGSIFSRIAMXPICDLLGPRIAIATLSLLTAPVILSTSLVSSPNSFIAIR

MDP0000131368 FSIPPLLAVIRNDLNLTDTDTGYAGTAAFVGSIVSRIAMGPICDLIGPRIAIATLSLLTAXIIXSTSLISSPNSFIAIR

**MFS**

MDP0000266497 FFTGFSLATFVSTQFWMSSMFSPPVVGTANGVAGGWGNLGGGATQLIMPVVFGFIRDIGAVKFTAWRIAFFIPALFQTL

MDP0000239537 FMIGFSLATFVSCQYWMSTMFNGKIIGLVNGTAAGWGNMGGGATQLLMPLMFDIIGRCGATPFTAWRIAFFIPGWFHII

MDP0000201530 FIAGFSLANFVANQFWMSCMFSGCVVGLANGFSXGWANMGSGVTQXVMPLIYSLIMSFNVXSFAAWRLAFIVPAVFQVV

MDP0000131368 FLAGFSLANFVANQFWMSSMFSGCVVGLANGFSAGWANMGSGVTQMVMPLIYSFIMSFDVPSSTAWRLAFFVPAIFQTV

**NNP**

MDP0000266497 SAFAILIFGQDMPDGNFYGLHKSGEKPKDKFSSVFYHGVTNYRGWILALTYGYCFGVELAVDNIIAEYFYDRFDLKLHT

MDP0000239537 TGIMVLTLGQDLPDGNLGALQKKGEVAKDQFSKVLWYAVTNYRTWIFVLLYGYSMGVELSIDNVIAEYFYDRFDLKLHL

MDP0000201530 TAXLVLMFGQDXPXGSYKXSQKVENKXKESLLSVISXGVKNYRAWILALTYAFSFGVELTTDNIIAQYFYDRFNVNLEV

MDP0000131368 TALLVLAFGQDLPSGSYKRSQKINNGSEESLLSVISNGAKNYRAWILALTYAFSFGVELTTDNIIAEYFYDRFGVNLEV

MDP0000266497 AGMIAASFGLANIVSRPGGGILSDVVAKKFGMRGRLWTLWVVQTTGGVLCVILGRVNSLVGSIVVMILFSVFVQGACGL

MDP0000239537 AGIIAASFGMANFAARPFGGWASDVAGRYFGMRGRLWTLWILQTLGGVFCICLGRANSLPLAVLAMILFSIGAQAACGA

MDP0000201530 AGIIAASFGMANFFSRPSGGLVSDRLARRFGMRGRLWGLWVAQTVAGLLCLLLGRVNSLWGSILVMCAFSVFVQAAAGL

MDP0000131368 AGXIAASFGMANFFSRPSGGLVSDKLAQRFGMRGRLWGLWVAQTVAGCLCLLLGRVNSLWGSILVMCAFSIFVQAAAGL

MDP0000266497 TFGVVPFVSRRSLGVISGMTGGGGNVGAVLTQLIFFKGSKYSKETGITLMGIMIICCTLPMTLIYFPQWGGMFCGPSKN

MDP0000239537 TFGVIPFISRRSLGIISGLTGAGGNFGSGLTQLVFFSTSAFSTASGLSWMGVMIVCCTLPVTLVHFPQWGGMFLPASKD

MDP0000201530 TFGVVPFVSKR-QAPNSHLTNG---------------------QKSITLRSVDNVLEWPMIVGADGNCW

MDP0000131368 TFGVVPFVSKRSLGXVSGITXGGGTMGAVVTQLLLFSGTEFSRQTSISLMGVMMLVCTLPVSLIYFPKWGGMFCRPSYG

MDP0000266497 --KATEEDYYMSEWSSKEKEKGFHQASVKFAENSRSERGKSDSVTRPSDEISPPHV

MDP0000239537 VEKSTEEFYYAAEWSEAEKQKGLHQGSLKFAENSRSERGRRVASVPTPPNTTPSHV

MDP0000201530

MDP0000131368 -----YGETNQYHLLQ

Additiional File 2. Alignment of full length apple High Affinity Nitrate Transporter genes.

Genes were identified by BLASTn analysis of the apple genome (FEMA-IASMA) using the original EST isolated from apple roots after two weeks at 45% of water saturation. The full length polypeptide ID from the genome is MDP0000239537. The other polypeptide IDs are discussed in the text. Protein IDs can be used to search the apple genome for further information regarding their location, prediction parameters and structure. MFS: major facilitator superfamily; NNP: nitrate/nitrite porter. Bases other than A, C, G, T in the nucleic acid sequence are translated into X by the translation programs.
