# Supplementary material for: Genes responding to water deficit in apple (Malus × domestica Borkh.) roots
Source: BMC Plant Biol. 2014 Jul 8;14:182. doi: 10.1186/1471-2229-14-182 (PMC4110548; doi:10.1186/1471-2229-14-182)
Supplement: Additional file 3 — Comparison of HAT2.4 promoter region from apple and Arabidopsis. Cis-elements identified by PLACE [57] and PLANTCare [59-69] are shown for the first 700 bases upstream of the translation start (atg) [63-70]. [file 1471-2229-14-182-S3.docx]

**A**

-710 AGGTGTCATTTTCAAATACATCTCACATGCATTTCTAATGCTCATAGAGG**TCATTACTAA**TCTCTTCTAA

-640 TCTTATATTTTATGGCCCATTTCTTACTAACTTTAACTCAATTCTTTGTGACTTAGGATATCATCTAACA

-570 TAGCCTCAATATGCGCTTCGGACGAGCAATTTCAAGACTCATGCAAATTCTTCGATTACAAGTGGGCAAA

-500 GTACAATGTTACAGATTGCATTATTAACCAATCAAAGTGGATCATTTACTAGTTGTATATATATAATTTA

-430 GGTTTATGAAGCCAAGCATTACTCCAAACAAACAATATTCAAATATAATTTCCTTTGACGTGTGCAAATT

-360 CCGGGCCGACTTAATTATTAGGAGTTCATTGACTTAGTTAATACTTGGTTGTCCCTAAGATGCAAGACAG

-290 TTGTTAATTAAC**CAGCTG**ATTGCAATTTTGAAGGGTTACTGTGATCCCTTGGGATATTCGATGAGACTTT

-220 TACTATTCATATCATCGTATGCGGCGAACTGGATCACACAAATGTGGAGAATCTTACTCTTGCCCGTTGC

-150 CCCTTCACAACCCTCCACAAC**GTCAACT**CCAAGCCCTATATAAATCCCACACTCCCTTTGTACCTTCCTC

-80 ACACAACACTACAATTCCGTTTAGTACTCTAATCAGCATCAAAATCCAAACCCCAAACCCCGAATTCCGA

-10 AGCCCCAAAA**atg**

**B**

-710 CTTCCTAAGCATAATTAGGGATCTGATTACATCATCACAACTTTAGTTTAATAAGTGAAAATCTATATAT

-640 TATTTTGTTTATTTCAATAACATTCTATTATATTAACAAAAAAAAAAAAAAAATCAAATGGAACCTTTGA

-570 ATCCATGCAAAGACATAGTCACATAGAGAGAGAGAGGACCCACCGGACTCGTTCACATGTATGAATATGG

-500 ATTGATAGTAAACTCTATGAGGATTTGCTGTATTAGACATGAAAAGAAATCGTTGTAAACAGAACCAGAA

-430 AGTAGTTTCTCGTTTGAGAAAAAAAAAAAAAAAAAAAACTTCCTAGATATATGGACAGTTTAGTAATATT

-360 ATATTGTTGATAAATACTAAATTGGAATATAAGTGAAAGTGAACCTTTGGG**ACGT**ATGACCAGGCTAAAT

-290 TCTCGTGTTTGCATGCTCGCGGCGAAAGTGAATATTCCATACTATTAATATAAAGACACAACTTGTATAA

-220 TTGTAATCAATCTCACATAAACAACTTTGAATCATTAATTATCGTTAAGAACTATACTATTATAGTATCA

-150 TTTACGTAAAACGAATCCCACTTATAGCCTTTTCGAATCTCCATCGGCTCTTACGAA**GTCAACT**TTCGTA

-80 TCTCTATATAAAACCATTCTTCTCCTTCTTGTTCCCTAAACCAAGACCACAAGAAAAAAACATAAAAAAA

-10 TATTCACAAA**atg**

Additional File 3. Comparison of *NRT2.4* promoter regions from apple and *Arabidopsis*. Only the first 700 bases upstream of the translation start (atg) are shown. *Cis*-elements were identified by PLACE [57] and PLANTCare [58]. A: Promoter region from *MdNRT2.4*; B: Promoter region from *AtNRT2.4*. **AAACAAA:** anaerobic induction [63]; TGACG: WRKY stress responsive binding element [64]; **A^A^/_C_GTCA** and **^G^/_A_GACTTTTC:** bZIP and NF-B binding sites, respectively [65]; **CAAGCATGCTTCTTGC:** consensus root-specific element [66]; **TATA box:** PolII binding; **TCATTACTAA:** wound-inducible element [36]; ACGTG/AT: G-box core element [34, 35]. An element of unknown function in the *Arabidopsis* *NRT2.4* gene promoter (**GTCAACT**) is also present in the *MdNRT2.4* promoter. A *cis*-element for hypoosmolarity-responsiveness [37] is identified by an oval. Dashed underlines indicate a MYB (WAACCA) binding site [67] and MYC core sequences (CANNTG); fuschia-underlined MYC element on the negative strand in A (CAACTG) is associated with drought response [68]. A CBF/DREB element [69-70] is boxed in A.
