# Supplementary material for: Genes responding to water deficit in apple (Malus × domestica Borkh.) roots
Source: BMC Plant Biol. 2014 Jul 8;14:182. doi: 10.1186/1471-2229-14-182 (PMC4110548; doi:10.1186/1471-2229-14-182)
Supplement: Additional file 4 — Alignment of apple TOM7 predicted polypeptides. Predicted polypeptides for the three apple TOM7 polypeptides and the EST isolated from drought-treated roots are aligned with the two predicted Arabidopsis TOM7 polypeptides [71]. [file 1471-2229-14-182-S4.docx]

MdTOM EST MASKISLKTKGKTP-AKGSKG---SEERSVAQFVKEWSTWTMKKAKVVTHYGFIPLVIIIGMNSDPKPQPSQLLSPV

MDP0000023053 MASKISLKTKGKTP-AKGSKG---SEERSVAQFVKEWSTWTMKKAKVVTHYGFIPLVIIIGMNSDPKPQLSQLLSPV

MDP0000694615 MASKISLKTKGKTP-AKGSKG---SDERSVAQSVKEWSTWTMKKAKVVTHYGFIPLVIIIGMNSDPKPQLSQLPKPQNFNFFA...

MDP0000878094 MASRISLKSKGKTP-AKPSKG---SEERSVAQSFKEWSTWALKKAKVVTHYGFIPLVIIIGMNSEPKPQLSQLLSPV

AtTOM7-1 MESTISLKVN-KGK-GKGSKGASSSDDKSKFDVVKEWTNWSLKKAKVVTHYGFIPLVIFVGMNSDPKPHLFQLLSPV

AtTOM7-2 MAAKSTLKIKGKAKPSKGSSSSSSSSASSKYKVFKDWTNWSLQKAKVATHYGFIPLIIIIGMNSDPKPHLFHLLSPV

Additional File 4. Alignment of apple TOM7 predicted polypeptides.

Predicted polypeptides for the three apple TOM7 polypeptides and the EST isolated from drought-treated roots are aligned with the two predicted Arabidopsis TOM7 polypeptides. The unique TOM7 domain characteristic of this superfamily [71] is highlighted in yellow. MDP000023053 is MdTOM7.1; MDP0000694615 is MdTOM7.2; MDP0000878094 is MdTOM7.3.
