# Supplementary material for: Genes responding to water deficit in apple (Malus × domestica Borkh.) roots
Source: BMC Plant Biol. 2014 Jul 8;14:182. doi: 10.1186/1471-2229-14-182 (PMC4110548; doi:10.1186/1471-2229-14-182)
Supplement: Additional file 5 — MdTOM7 promoter regions. Sequences approximately 800 bases upstream of the translation start (atg) were analyzed by PLACE, PlantPAN and PlantCare [72][67]. [file 1471-2229-14-182-S5.docx]

MdTom7.1

-820 TGACGAGGCACCCACCTTAGGTTGATCATATCCCTAATGA

-780 CGTTCCGTAGACGTTTCATTTGACTTTGCATTTCTCACGCATTTTYCCTTAGCCTATGGGTTTTGTCCCT

-710 CCTACTCGGGTTTTACCATAGTCATTGGGTTTTTTGTGAGACTTACTTCCATATGTAAGTTCCTACCTTA

-640 TTTAAGAATAGYGTGTTCATAGAATCAGTGCCRACAAGTTKACTTCCTCAATTCTACATGATGCCGAATC

-570 TACTTAGTATTTACACACTCAAAGGGGGTGTTATAAACTTCTTATTTAAGTGTGATTGTGTAAATCCTAA

-500 ATTAGATTTGATTCAAGTTATTCCTTCCTATTAGGACTTGTATTCCTKGGAGGAGAAKGATTTYTTCCCT

-430 YCCTTATTACTATAAATAAAGGCATTGTGTAGGGGAAAAACACATCTTATACAYAATCCTACAAACACAT

-360 CTCTCTYTTTTCACTTTGTATCGCCGGCCCTCTCTCCCTTGTCAGTTAAATATATGCCACAACGATAACT

-290 AAATTGTCATGTCTTTTAATTTTGTTTAGCTTTGATGAGAGGGTTAGAAAAATAATTTTATARAGTTTTG

-220 ATTGACAAATRGAGTTTTATAATAGGTAGTCGCTTAGATGATTTAATTTTTTTCTTTTGTGAGAGARAAA

-150 AAAAAACGACGGCTGTGATGCACGYCACACACAAAGTGGWGTCTGGACACTTGTTAGGGCCCCAACCCCC

-80 AAACAAGCGGATCAATATAATTAGGGTTTGGAGAGAGAGAGAGGAGCGCAGACTCACACGAGCTCCAGAA

-10 GAAAATTTCAatg

MdTom7.2

-820 TCTAACCTTGTTTATACCATATTTAGGGCCTCGTATTTAG

-780 ATCTCGTACAAATACTCAGGGGACTTAAATGTAATTATGTGATAAAGAAAGGGGCAAATATGTAATAAGT

-710 GAGGAGTCCTTATTCTATAAAAGGACCCCTCACCCTCACAATTAGAGAAGTCCAATTCCTAGGCCATCAG

-640 AGACTTCACTCTCTCCCTCAGAGGCTATGAATCTCTTTCCCTCACTCCCCTCACATCTCAGATAAATACA

-570 TAATCAGTGTGGACGTAGCCCAAACCTTGGGGTGAACCACGATACATCTTGTGTTATTTACATTTCTTGC

-500 AGATTCACGGTTGGATTTACGTTGTTCCAATACCTCCGGTTTTGTGCATCAACATTTGGCGCCGTCTGTG

-430 GGAATCAATACGAAAAGTTGTGTCAGTTCTCTTTCATTTTTTCATCTCCACCTTGAAACTGCAAAAACCC

-360 AAGAACCCAAAAATTTCCCAGAAAACCCACGGAGCCACCTCCTCTATGACTCAATCTCTCTCTGTAACTG

-290 CACAGACCACGTGTCTGGCCTCCCTCTCTCTTCTTCTTCTTCGTTTTCTCTCTCTGCCAAAGCTCGAAAG

-220 TTTCAATGATTGGGGAAGCCAGGTTCACGATCAGAATCGTCGACCAAAGCCGACCGTAAGTTCGAAAAGA

-150 AGCTCGAATTTTATGCTAAGGTTAGAGATGCAGTTTTTTCCTTAGGTGCACAGAAGGCTATTGTTAAGAG

-80 TTAAGAGCAATTGTTCCTAACAGTCATAACTATAAAGACAATGGTAACAATAACAGCAATAACTCCAGAA

-10 GAAAATTTCAatg

MdTom7.3

-820 GCGTTCAACGCAACATCAACACATTTTACTCCCCAACCAA

-780 GCTCGGCCGACGAGTTGGCACGCCCCACACACAACCAAAGAACGTAGTTAGCTTATTAATTACTCGGCCT

-710 GCGCGCCACCTAGGCTTGATAGTTTTTAGGATCAACACACTCTTAGGTCTATCGTTTGACTATCTGATTG

-640 TTGTATGTGTGGCACTTTTTTTTCATATCTTGAAGTTTTCTCCCAAGTGATTTTCTTCATGATTTTACTG

-570 AGATCCACATCTTTAGAGTACACTGTTTGTCTATTTGGCTCATTATTTTGTATTGCATTGGATATGTCGT

-500 TAGGCTGATGAAATTTGTTTGTATTTTTGTTTTCTTTTGCCCCGTGATAGCTTATTTAGGCAGAATGTCA

-430 TTTTAAGACGCAACTTTTATTTTTGATGGTCACGTTTAATTTTTACGCAATCGTTTGTATTCTTTATTGG

-360 TGTTTGAATAAAAAATTTTGAAAAAAAAATCAAAACACCCAAGTTTTTATTAACAAATATGATTTGATTC

-290 TAAAAAATCAAAATCCAATTGTTTTTTTTTTATTGGTGTTTGAATAAAGCATCTTTTTTTGAAAGAAAAA

-220 AAAATCAAAACACCCATCTTTTCTTCCACCAACCTACTAATAGCATGAATTAGATTTTTTTTTTTTTCTT

-150 TTCTGAGAGATAAATGTTAGAATTTTGTGGAAGAAACGAAAAGGAAAAAAACGACAATTTATATCCCCAA

-80 CCACAAAGCAGTCAGATCAATATAATTAGGGTTTTAGAGAGAGGCGCGCAGACTCGCACAAACCCTAGAA

-10 GCAAATTTCAatg

Additional File 5. MdTOM7 promoter regions.

Sequences approximately 800 bases upstream of the translation start (atg) were analyzed by PLACE, PlantPAN and PlantCare. The probable consensus TATA box is underlined. A pyrimidine box (TTTTTTCC) associated with GA induction is marked in yellow along with other elements required for GA response (72). The ABA response element (ACGTGTC) is highlighted in magenta. Blue highlight indicates the presence of a CRT/DREB element (RCYGAC), and MYC binding sites (CANNTG) are marked in gray (67).
